# Successful surgical retrieval of Celt ACD® vascular closure device embolised in the tibioperoneal trunk

**DOI:** 10.1186/s42155-018-0013-5

**Published:** 2018-08-07

**Authors:** Qusai Aljarrah, Ma’moon Al-Omari, Kawthar Qader, Jozef Oweis, Ahmad Althaher

**Affiliations:** 1 Vascular Surgery, Jordan University of Science and Technology, Irbid, Jordan; 2 Interventional Radiology, Jordan University of Science and Technology, Irbid, Jordan; 3 King Abdullah University Hospital, Irbid, Jordan; 4 Jordan University of Science and Technology, Irbid, Jordan

**Keywords:** Vascular closure device, Foreign body embolism, Tibioperoneal trunk, Celt ACD®

## Abstract

**Background:**

This report presents a case of surgical retrieval of a Celt ACD® vascular closure device (VCD) situated in the tibioperoneal trunk, following a failed attempt at deployment. Existing literature mostly recommends an endovascular approach when attempting to retrieve embolised VCDs.

**Case presentation:**

A 55 year old male presented with right sudden right lower limb pain and numbness 1 week following a successful left retrograde superficial femoral artery (SFA) angioplasty. Computed tomography (CT) angiogram revealed that the Celt ACD® VCD had embolised in the right tibioperoneal trunk. An endovascular approach was initially attempted to retrieve the VCD; however, this was unsuccessful due to the small diameter of the target artery. Due to the failure of the endovascular approach, surgical exploration of the right tibioperoneal trunk was undertaken, which led to the successful retrieval of the embolised VCD.

**Conclusion:**

The case presented herein demonstrates the critical need for swift and decisive surgical exploration of patients with suspected embolisation of Celt ACD® devices in smaller distal arteries. Our experience has led to the recommendation that, due to the sharp edges of the Celt ACD® accompanied with the small diameter of the occluded vessels, surgical exposure and retrieval is the safest option if endovascular retrieval is unsuccessful.

## Background

The use of vascular closure devices (VCDs) have become prominent in endovascular surgery. Their application has allowed for faster achievement of hemostasis at the access site, early recovery following intervention and early mobilization (Biancari et al. 2010). However, reported complications can vary from infection, vascular dissection, vascular occlusion and distal embolisation (Biancari et al. 2010). Celt ACD® (Vasorum Ltd. Dublin, Ireland) is a distinctive VCD that achieves hemostasis through the delivery of a stainless steel plug, anchored on both sides of the arterial wall by extendable wings. This case illustrates the surgical retrieval of the device following a complication of distal embolisation.

## Case presentation

A 55 year old male presented to our hospital with critical left lower limb ischemia with associated great toe ulceration. Computed tomography (CT) angiogram revealed a Trans-Atlantic Inter-Society Consensus (TASC) A lesion in the left superficial femoral artery (SFA) with patent run-off vessels. The patient underwent left retrograde SFA angioplasty via an ultrasound guided right common femoral artery puncture. Drug eluting balloon angioplasty was performed for the SFA lesion and a confirmatory angiogram revealed satisfactory results with patent run-off vessels. Access site control was attempted utilizing the Celt ACD® device. Unfortunately, continuous bleeding from the access point was noticed. Therefore, manual compression was applied for 10 min. Post procedure, the patient had palpable pedal pulses and satisfactory access site appearance. His discharge medications included dual anti-platelet therapy and statins.

**Fig. 1:**
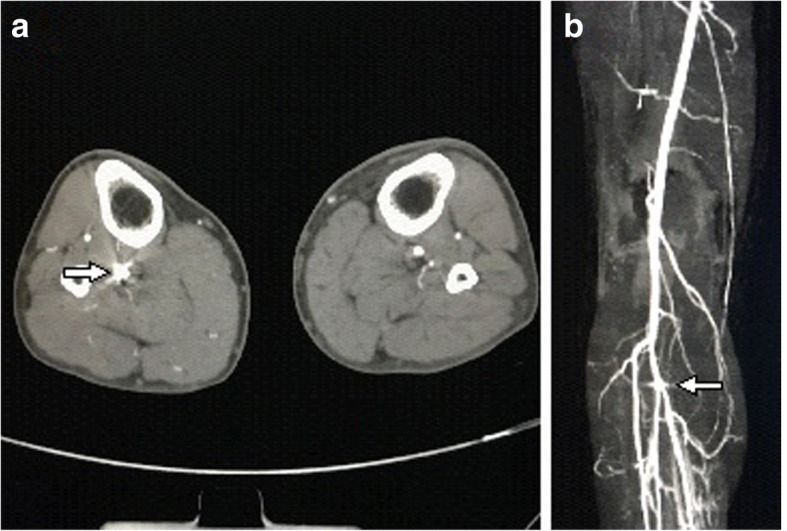
**a** Computed tomography (CT) angiogram axial view of the lower limbs. The arrow points to an artifact occluding the right TPT. **b** CT angiogram coronal view of the right lower limb. The arrow points to an artifact occluding the right TPT

One week following discharge, the patient developed sudden pain and numbness in his right foot with short distance calf claudication. On clinical evaluation, the patient denied any symptoms of critical limb ischemia. Physical examination confirmed an unremarkable access site, palpable femoral and popliteal pulses, but absent pedal pulses. Consequently, a lower limb CT angiogram was conducted and revealed a metallic artifact in the distal popliteal artery consistent with VCD embolisation (Fig. 1a and b).

**Fig. 2:**
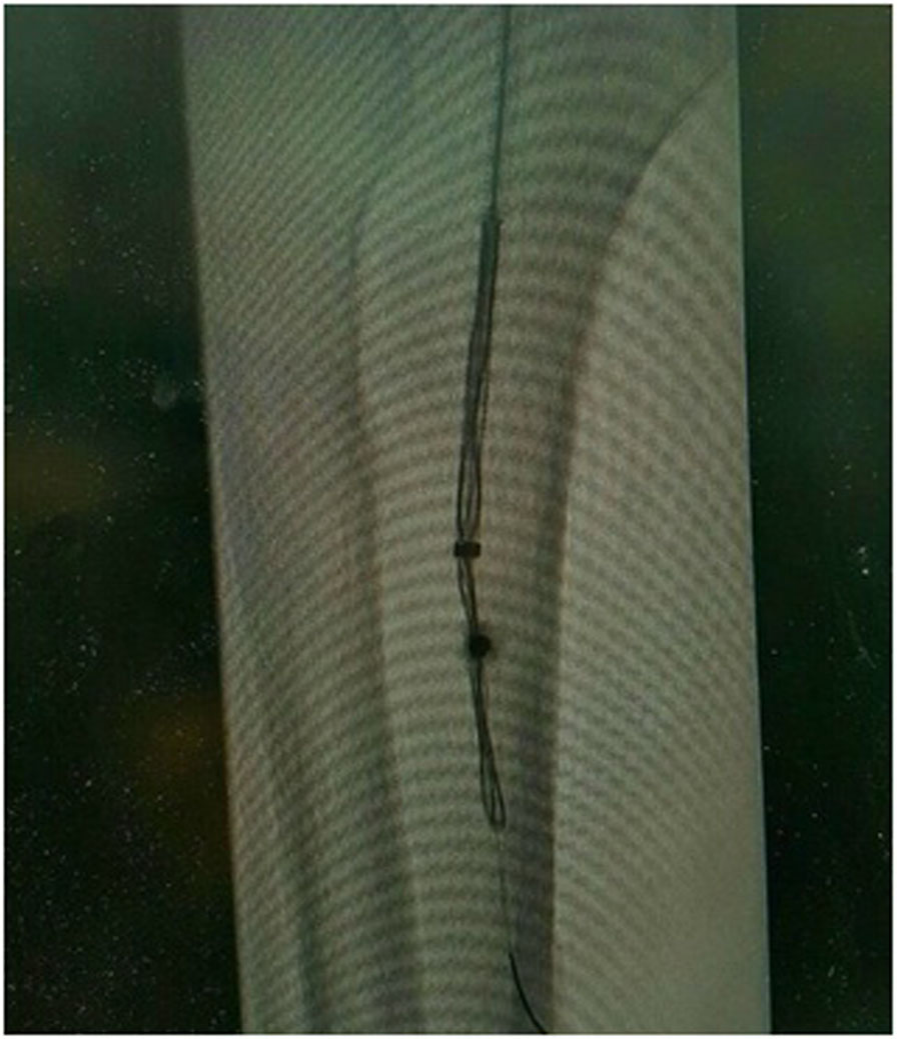
Attempting an endovascular retrieval of the closure device using a snare (Indy OTW™ Vascular Retriever, 8Fr, .35 mm, 100 cm)

Endovascular retrieval of the closure device was attempted through the right antegrade common femoral artery using a snare (Indy OTW™ Vascular Retriever, 8Fr, .35 mm, 100 cm; Fig. 2). Unfortunately, the attempt was unsuccessful and the patient developed severe spasm of the popliteal artery.

**Fig. 3:**
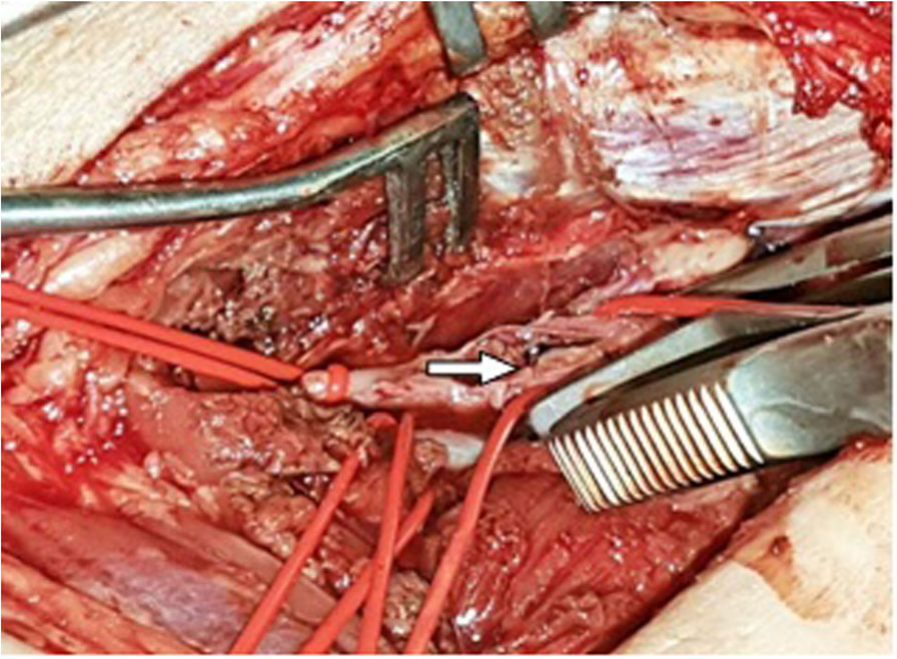
Surgical exploration of the right TPT. The arrow points towards a metallic artifact revealed in the right TPT

The following day, the patient’s symptoms worsened and he developed rest pain. Therefore, we opted for surgical exploration of the distal popliteal artery and tibioperoneal trunk (TPT), which revealed a thrombus in the explored arteries with the closure device occluding the TPT bifurcation. Selective embolectomy of the anterior tibial artery and TPT with device retrieval was successfully accomplished (Fig. 3). Dacron patch was used for arteriotomy closure. On table doppler ultrasound was done and demonstrated good signals in the patch and crural vessels.

The patient’s post-operative course was unremarkable; his pedal pulses were restored and he was discharged 3 days following the procedure on dual anti-platelet therapy. At 3 months follow up, the patient was doing well with completely healed wounds and palpable pedal pulses in both lower limbs.

## Discussion

Before the introduction of VCDs in the 1990s, the benchmark for vascular access-site control was manual compression. VCD are classified into three major categories; plug-based, suture-mediated, and clip-based (Bechara et al. 2010)*.*The use of VCDs have been favoured over manual compression because of their capacity to reduce the time to hemostasis and promote early patient mobilization (Bechara et al. 2010; Bangalore et al. 2009). However, the safety and efficacy of these devices, has come under scrutiny due to their reported association with vascular access-site complications including infection, bleeding and limb ischemia (Biancari et al. 2010; Bechara et al. 2010; Bangalore et al. 2009).

The incidence of such vascular complications has been found to be higher in cases of device failure (Bangalore et al. 2009). Bangalore et al. reports that device failure is significantly associated with elderly patients and those suffering from diabetes or peripheral artery disease (Bangalore et al. 2009). Additionally, suture-mediated VCDs in particular are reported to be associated with a higher risk of failure compared to other types of devices (Bangalore et al. 2009). On the other hand, ischemic complications have been found to be more common in devices which employ an intraluminal component such as Celt ACD® and Angio-Seal® (St. Jude Medical, Inc., St. Paul, Minn). This is due to the higher risk of distal embolisation of the intraluminal component (Suri et al. 2015).

The Celt ACD® is a novel stainless steel VCD with both intraluminal and extraluminal components. It has several potential advantages over other VCDs including a reduced time to hemostasis and a faster deployment utilising the existing procedural sheath (Jan et al. 2013). Its distinctive metallic structure renders it fluoroscopically detectable, aiding in precise localization of the device in cases of mal-deployment. In addition, Celt ACD® can be utilized in narrower vessels due to its smaller size compared to other VCDs (Jan et al. 2013). When compared to Angio-Seal® in particular, Celt ACD® has demonstrated lower patient discomfort and a reduced incidence of late minor bruising (Cahill et al. 2014).

However, the sharp edges of the intraluminal component might cause serious intimal injury and consequent thrombus formation in the event of distal embolisation of VCDs. In cases of distal embolisation of VCDs, open surgical embolectomies have conventionally been carried out to retrieve the mal-deployed device (Prabhudesai and Khan 2000; van der Steeg et al. 2009). However, cases of successful retrieval of embolised Angio-Seal® devices through endovascular approaches have been reported (Suri et al. 2015; Jud et al. 2017). The successful endovascular retrieval of a distally embolised Celt ACD®, following primary device failure, was reported by Cahill et al. (Cahill et al. 2013). The device was visualised fluoroscopically and found to be situated in the common femoral artery. In order to avoid the subsequent consequences of vessel occlusion, the embolised device was retrieved through an endovascular approach using a triple-looped EN snare (Merit Medical, South Jordan, UT).

In our case, the Celt ACD® embolised to a more distal location and was found to be situated in the TPT. Endovascular retrieval was attempted utilising a snare but this was unsuccessful due to the small diameter of the target artery. Moreover, endovascular manipulation and intimal injury caused by the device led to subsequent thrombosis. Consequently, open surgical exploration led to the successful retrieval of the mal-deployed device.

## Conclusion

To conclude, we strongly concur with Kalapatu et al. that the site of occlusion dictates the method used for retrieval (Kalapatapu et al. 2006). Early recognition of device failure is crucial to prevent distal embolisation. Our recommendation on the management of embolised Celt ACD® devices in distal arteries is that open surgical retrieval remains the safest option if endovascular retrieval is unsuccessful. Furthermore, endovascular retrieval of the device via a long guiding sheath is essential to avoid intimal injury if endovascular attempt was committed. Finally adequate operator training is vital to reduce the risk of mal-deployment.

## Abbreviations

CT: Computed tomography
SFA: Superficial femoral artery
TASC: Trans-atlantic inter-society consensus
TPT: Tibioperoneal trunk
VCDs: Vascular closure devices
